# Evaluating Lung Changes in Long COVID: Ultra-Low‐Dose vs. Standard‐Dose CT Chest

**DOI:** 10.3389/bjbs.2024.13385

**Published:** 2024-09-10

**Authors:** Shritik Devkota, Mandeep Garg, Uma Debi, Sahajal Dhooria, Ashish Dua, Nidhi Prabhakar, Saumya Soni, Muniraju Maralakunte, Ajay Gulati, Tarvinder Singh, Manavjit Singh Sandhu

**Affiliations:** ^1^ Department of Radiodiagnosis and Imaging, Post Graduate Institute of Medical Education and Research (PGIMER), Chandigarh, India; ^2^ Department of Pulmonary Medicine, Post Graduate Institute of Medical Education and Research (PGIMER), Chandigarh, India

**Keywords:** radiation dose, COVID-19, long COVID, PASC, post-acute sequelae of COVID-19, ULDCT chest, Ultra-low-dose CT chest

## Abstract

**Background:**

Frequent chest CTs within a short period during follow-up of long COVID patients may increase the risk of radiation-related health effects in the exposed individuals. We aimed to assess the image quality and diagnostic accuracy of ultra-low-dose CT (ULDCT) chest compared to standard-dose CT (SDCT) in detecting lung abnormalities associated with long COVID.

**Methods:**

In this prospective study, 100 long COVID patients with respiratory dysfunction underwent SDCT and ULDCT chest that were compared in terms of objective (signal-to-noise ratio, SNR) and subjective image quality (image graininess, sharpness, artifacts, and diagnostic accuracy along with the European guidelines on image quality criteria for CT chest), detection of imaging patterns of long COVID, CT severity score, and effective radiation dose. Additionally, the diagnostic performance of ULDCT was compared among obese (BMI≥30 kg/m^2^) and non-obese (BMI<30 kg/m^2^) subjects.

**Results:**

The mean age of study participants was 53 ± 12.9 years, and 68% were male. The mean SNR was 31.4 ± 5.5 and 11.3 ± 4.6 for SDCT and ULDCT respectively (p< 0.0001). Common findings seen on SDCT included ground-glass opacities (GGOs, 77%), septal thickening/reticulations (67%), atelectatic/parenchymal bands (63%) and nodules (26%). ULDCT provided sharp images, with no/minimal graininess, and high diagnostic confidence in 81%, 82% and 80% of the cases respectively. The sensitivity of ULDCT for various patterns of long COVID was 72.7% (GGOs), 71.6% (interlobular septal thickening/reticulations), 100% (consolidation), 81% (atelectatic/parenchymal bands) and 76.9% (nodules). ULDCT scans in non-obese subjects exhibited a significantly higher sensitivity (88% vs. 60.3%, p < 0.0001) and diagnostic accuracy (97.7% vs. 84.9%, p < 0.0001) compared to obese subjects. ULDCT showed very strong correlation with SDCT in terms of CT severity score (r = 0.996, p < 0.0001). The mean effective radiation dose with ULDCT was 0.25 ± 0.02 mSv with net radiation dose reduction of 94.8% ± 1.7% (p < 0.0001) when compared to SDCT (5.5 ± 1.96 mSv).

**Conclusion:**

ULDCT scans achieved comparable diagnostic accuracy to SDCT for detecting long COVID lung abnormalities in non-obese patients, while significantly reducing radiation exposure.

## Introduction

The COVID-19 pandemic has precipitated an unprecedented global health crisis, impacting millions. Extensive research has revealed that a substantial proportion of individuals continue to experience persistent health issues following recovery from acute COVID-19 infection, often with manifestations across multiple organ systems [1–4]. Numerous terms (like long COVID, chronic COVID, long-haul COVID, post-acute sequelae of COVID-19 (PASC), post-COVID syndrome, etc.) have been used to describe this condition [2–6], with Long COVID and PASC emerging as the preferred terms in the recent scientific literature [2–4, 6].

Lungs are the most commonly affected organ by COVID-19 and a significant number of patients continue to have prolonged respiratory issues that warrant close observation with appropriate investigations. A recent meta-analysis has shown that lung parenchymal abnormalities are seen in the pooled frequency of 43.5% on one-year imaging follow-ups [7]. CT chest remains an indispensable follow-up imaging tool with many patients often requiring multiple repeat scans to monitor the disease evolution [2, 5, 7–9]. Since CT involves the use of harmful ionizing radiation that carries the risk of producing double-stranded DNA breaks and heritable genetic mutations; such repeated CT acquisitions can lead to increased cumulative dose with resultant increased risk of radiation-induced health hazards in the exposed individuals [10–13]. The extended imaging follow-ups for the long COVID patients necessitate balancing the benefits of diagnosis with the potential long-term risks of ionizing radiation exposure.

The routinely performed standard-dose CT (SDCT) chest delivers an effective radiation dose between 4-7 millisievert (mSv) to the patient [14, 15]. However, with the recent advances in CT hardware and software, radiation exposure to patients can be significantly contained by using newer CT techniques like low-dose CT (LDCT) and ultra-low-dose CT (ULDCT). The effective dose received by a patient undergoing CT chest with LDCT and ULDCT protocols is in the range of 1–4 mSv, and <1 mSv, respectively; and it can be a futuristic and pragmatic way to minimize the health effects of ionizing radiation [16, 17]. The only apparent limitation of these low dose CT techniques is of compromised image quality, that can affect the diagnostic accuracy; and more research is needed in this direction.

There have been few studies on the utility of LDCT and ULDCT in acute COVID-19 pneumonia that have shown promising results [18–24]. However, to the best of our knowledge, only one study to date has been published in the English literature [25] evaluating the utility of ULDCT in patients with Long COVID/PASC. We share our experience in evaluating and comparing ULDCT chest and SDCT chest in terms of image quality and diagnostic accuracy for detecting the lung changes long COVID. With radiation exposure, comparable to only a couple of chest X-rays, ULDCT chest may prove to be a much safer follow-up imaging tool in patients with long COVID.

## Materials and Methods

### Study Design

This was a prospective, observational study, approved by the Institutional Ethics Committee (Reference No: NK/7642/MD/816). Informed written consent was obtained from all study participants. Patients were excluded if they had pre-existing lung parenchymal diseases, a known history of pulmonary infection (other than COVID-19) or malignancy, were under 18 years of age, or declined to provide consent. Patients who recovered from moderate to severe acute COVID-19 pneumonia and visited our hospital between July 2021 to December 2022 with complaints of respiratory symptoms at least 12 weeks after the initial onset of symptoms were finally enrolled in this study. The demographic and clinical details of the included study subjects were collected from the hospital health records.

### CT Chest Acquisition Protocol

The scans were acquired on a 256-slice CT scanner (Philips Brilliance iCT256; Koninklijke Philips N.V., Netherlands). The enrolled participants underwent both SDCT and ULDCT scans consecutively. The kilo voltage peak (kVp) for SDCT and ULDCT scans were 120 kVp and 80 kVp respectively; while tube current exposure (mAs) for SDCT was automatic exposure control (AEC) modulated tube current, and for ULDCT, it was 25 mAs with fixed tube current. The rest of the image acquisition parameters were kept similar in both scans. The details of acquisition protocol for SDCT and ULDCT chest are summarised in Supplementary Table S1.

### Radiation Dose Calculation

The dose report was used to derive the CT dose index for volume (CTDI_vol_) and the dose-length product (DLP). DLP was multiplied with conversion factor (k) of 0.014 for thoracic imaging, as provided by International Commission on Radiological Protection (ICRP) 103, to calculate the effective radiation dose [26].

### Image Analysis

The scans were assessed and evaluated by 2 experienced chest radiologists (M.G. and U.D.) with 24 and 13 years of experience respectively and who were blinded to the clinical details of the patients. In a blinded design to minimize bias, radiologists first independently interpreted all ULDCT scans, followed by data entry. Subsequently, the ULDCT scans were masked to prevent recall bias, and finally, the radiologists evaluated a randomized selection of SDCT scans after 10 days. The findings recorded were - dose indices, image quality (both objective and subjective), pulmonary parenchymal abnormalities, and CT severity score. Any disagreement in the findings between the two observers was resolved by discussion and with mutual consensus. The data obtained from the analysis of ULDCT and SDCT scans were compared, keeping SDCT as the reference standard.

The objective image quality was assessed by obtaining image noise and determining the signal-to-noise ratio (SNR). The region of interest (RoI) of size ∼0.5 cm^2^ was kept in the tracheal lumen just above the carina. Care was taken that RoI did not touch the tracheal walls. Image noise is the standard deviation of the attenuation, while SNR is the ratio of mean attenuation to the standard deviation of attenuation.

The subjective image quality was determined by using European guidelines on image quality criteria for CT chest [27] along with 4 other parameters, viz. image graininess, image sharpness, artifacts affecting the image quality, and the diagnostic confidence for labelling a pulmonary finding as being present or absent. All the image findings were scored on a 3-point Likert scale. The image graininess was scored as 1 (no or minimal graininess), 2 (acceptable - low levels of image graininess, but images interpretable), or 3 (unacceptable – likely to misinterpret/miss imaging findings). Image sharpness was scored as 1 (sharp images), 2 (average sharpness, but images interpretable), or 3 (blurry images - likely to misinterpret/miss imaging findings). Artifacts were assessed as 1 (no artifacts), 2 (few artifacts present, but images interpretable), or 3 (artifacts present and likely to misinterpret/miss imaging findings). Also, the diagnostic confidence for labelling a pulmonary finding as being present or absent was evaluated as 1 (excellent), 2 (moderate), or 3 (poor).

The major imaging abnormalities assessed on ULDCT and SDCT were ground glass opacities (GGOs), consolidation, mosaic attenuation, traction bronchiectasis, septal thickening/reticulations, honeycombing, pleural thickening, nodules and subpleural cysts. Additionally, the presence of effusion (pleural or pericardial), hydropneumothorax and lymphadenopathy was also recorded.

CT severity of lung involvement was assessed by using a semiquantitative scoring system as suggested by Pan et al. [28] which ranged from 0 (no involvement) to 25 (maximum involvement). The CT severity scores of SDCT and ULDCT scans were also compared.

Further, the body mass index (BMI) was recorded for each patient and based on BMI data, the study subjects were divided into 2 groups – “obese” and “non-obese.” Subjects were labelled as “non-obese” if their BMI was <30 kg/m^2^ and “obese” if BMI was ≥30 kg/m^2^. Non-obese patients were further classified into three categories: underweight (BMI <18.5), normal weight (BMI 18.5–24.9), and overweight (BMI 25–29.9). Similarly, obese patients were categorized into class I (BMI 30–34.9), class II (BMI 35–39.9), and class III (BMI ≥40). The overall sensitivity, specificity, and diagnostic accuracy of ULDCT scans were also compared among the obese and non-obese groups.

### Statistical Analysis

The entire data was entered in Microsoft Excel spreadsheet and the final analysis was carried out using SPSS version 25.0. Mean ± standard deviation was used to express continuous variables. Student t-test was the parametric test used to analyse variables following normal distribution. The chi-square test and Fisher’s exact test were the non-parametric tests used to analyse variables that do not follow normal distribution. Sensitivity, specificity, positive predictive value (PPV), negative predictive value (NPV), and diagnostic accuracy of ULDCT for detecting imaging abnormalities were calculated, keeping SDCT as the reference standard. K (kappa) values were used to interpret the strength of agreement. Pearson’s correlation coefficient (r) was used for comparing CT severity scores of SDCT and ULDCT scans, and wherever applicable, a p-value <0.05 was deemed statistically significant.

## Results

### Enrolment and Baseline Demographics of Patients

**FIGURE 1:**
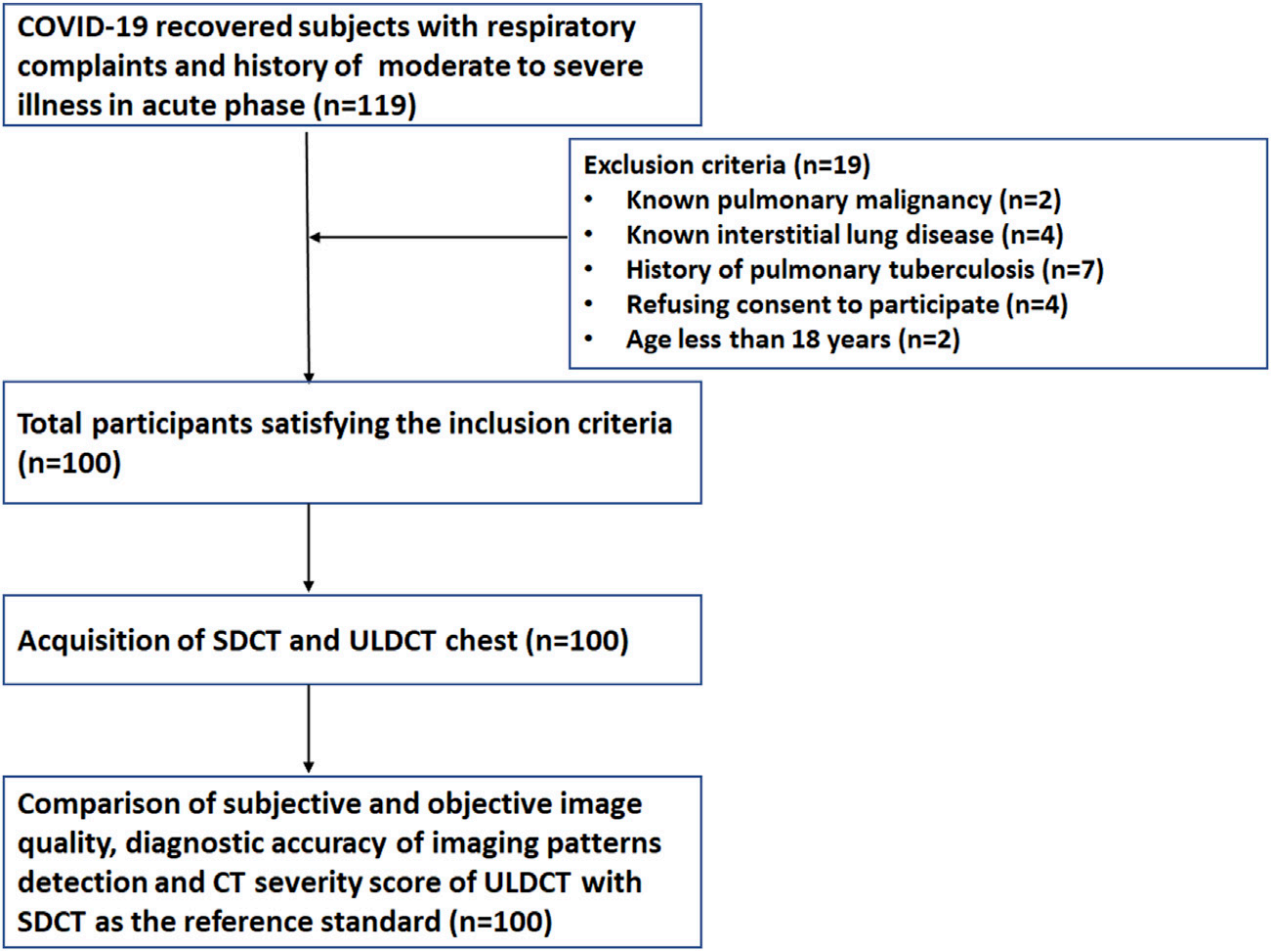
Flow design of the study participants.

Of the 119 initially identified participants, 19 were excluded from the study due to a history of pulmonary malignancy (n = 2), interstitial lung disease (ILD) (n = 4), pulmonary tuberculosis (n = 7), refusal to provide informed consent (n = 4), or age below 18 years (n = 2). Consequently, a total of 100 participants (68 male, 32 female) were enrolled in the study. (Figure 1). The mean age of study participants was 53 ± 12.9 years and 21% were grouped in the obese category based on their BMI. The mean duration between the onset of initial symptoms and acquisition of CT scan during the study period was 122.7 ± 54.4 days. The demographic data, clinical details, and BMI of the enrolled patients have been summarized in Table 1.

**TABLE 1:** Demographic, BMI, clinical laboratory details and duration from discharge to CT scan of the study subjects (n = 100).

Patient Characteristics	Frequency
Demographics	
Age (years)	53 ± 12.9 (mean ± S.D)
Gender	
Male	68 (68%)
Female	32 (32%)
BMI (kg/m2)	
Non-obese (<30)	79 (79%)
<18.5 (underweight)	5 (5%)
18.5-<25 (Normal)	56 (56%)
25-<30 (Overweight)	18 (18%)
Obese (≥30)	21 (21%)
Class I (30–34.9)	13 (13%)
Class II (35–39.9)	6 (6%)
Class III (≥40)	2 (2%)
Clinical features	
Fever	6 (6%)
Cough	40 (40%)
Dyspnea	54 (54%)
Myalgia	18 (18%)
Fatigue	58 (58%)
Comorbidities	
Diabetes	22 (22%)
Hypertension	14 (14%)
Asthma	4 (4%)
Coronary artery disease	4 (4%)
COPD/history of smoking	12 (12%)
Laboratory data	
Anaemia	18 (18%)
Leucocytosis	8 (8%)
Thrombocytopenia	6 (6%)
Duration from	Days
Onset of initial symptoms to CT scan	122.74 ± 54.42 (mean ± S.D)

Abbreviations: BMI, body mass index; COPD, chronic obstructive pulmonary disease; CT, computed tomography.

### Dose Indices and Image Quality Assessment

The dose indices and image quality assessment have been detailed in Supplementary Table S2. The mean effective radiation dose with ULDCT was 0.25 ± 0.02 mSv with net radiation dose reduction of 94.8% ± 1.7% (*p < 0.0001)* when compared to SDCT (5.5 ± 1.96 mSv). The mean SNR of SDCT and ULDCT scans were 31.4 ± 5.5 and 11.3 ± 4.6 respectively and it was statistically significant (p < 0.0001). There was minimal or no image graininess in 82% of the ULDCT scans. Sharp images were observed in 81% of the ULDCT scans. Artifacts were present in 13% of the ULDCT scans, of which 3% were likely to misinterpret/miss imaging findings. Images with unacceptable levels of graininess and poor diagnostic confidence were seen in 7% and 9% of the ULDCT scans. Only two of the ULDCT scans done in non-obese subjects had unacceptable level of image graininess and images of poor diagnostic confidence.

Sharp visualization of segmental bronchi and lung parenchyma was seen in 84% and 89% of the ULDCT scans respectively, while the border between the pleura and the thoracic wall, and pleuro-mediastinal border was sharply delineated in 85% and 86% of scans. Sharp reproduction of the trachea and major bronchi was seen in 92% of the ULDCT scans. 100% of the ULDCT scans satisfied the rest of the image quality evaluation criteria as per European guidelines (Supplementary Table S3). The ULDCT scans with compromised image quality were seen only in the “obese” group of subjects.

### Imaging Findings

**FIGURE 2:**
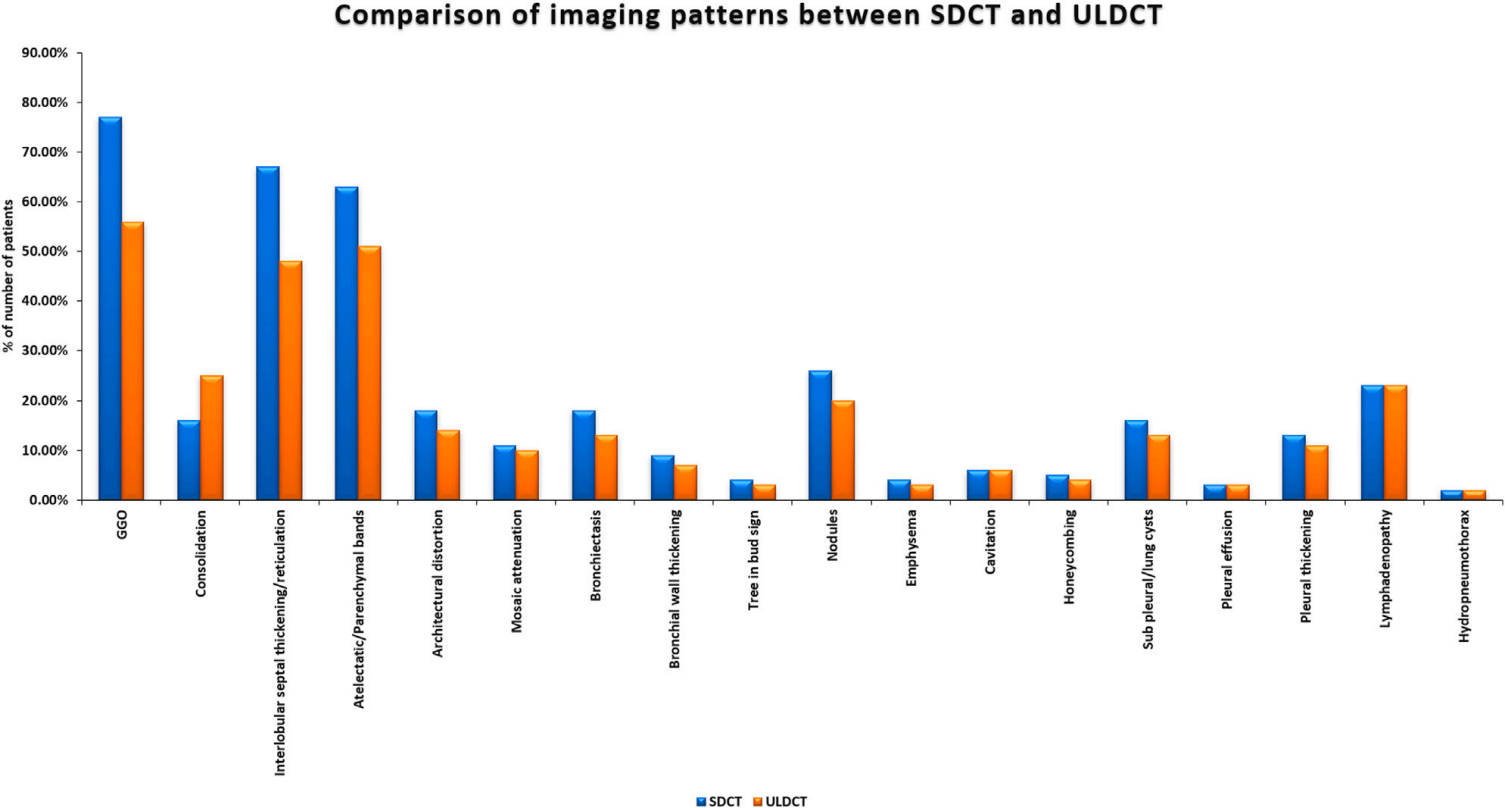
Bar diagram showing imaging patterns in long COVID as detected by Standard-dose CT (SDCT) and Ultra-low dose CT (ULDCT).

All (100%) patients enrolled in our study exhibited at least one relevant radiological finding. The common imaging abnormalities observed on SDCT and ULDCT scans (reference standard) (shown as a bar diagram in Figure 2) in order of their prevalence were – GGOs (77% vs. 56%), interlobular septal thickening/reticulation (67% vs. 48%), atelectatic/parenchymal bands (63% vs. 51%) and nodules (26% vs. 20%).

**FIGURE 3:**
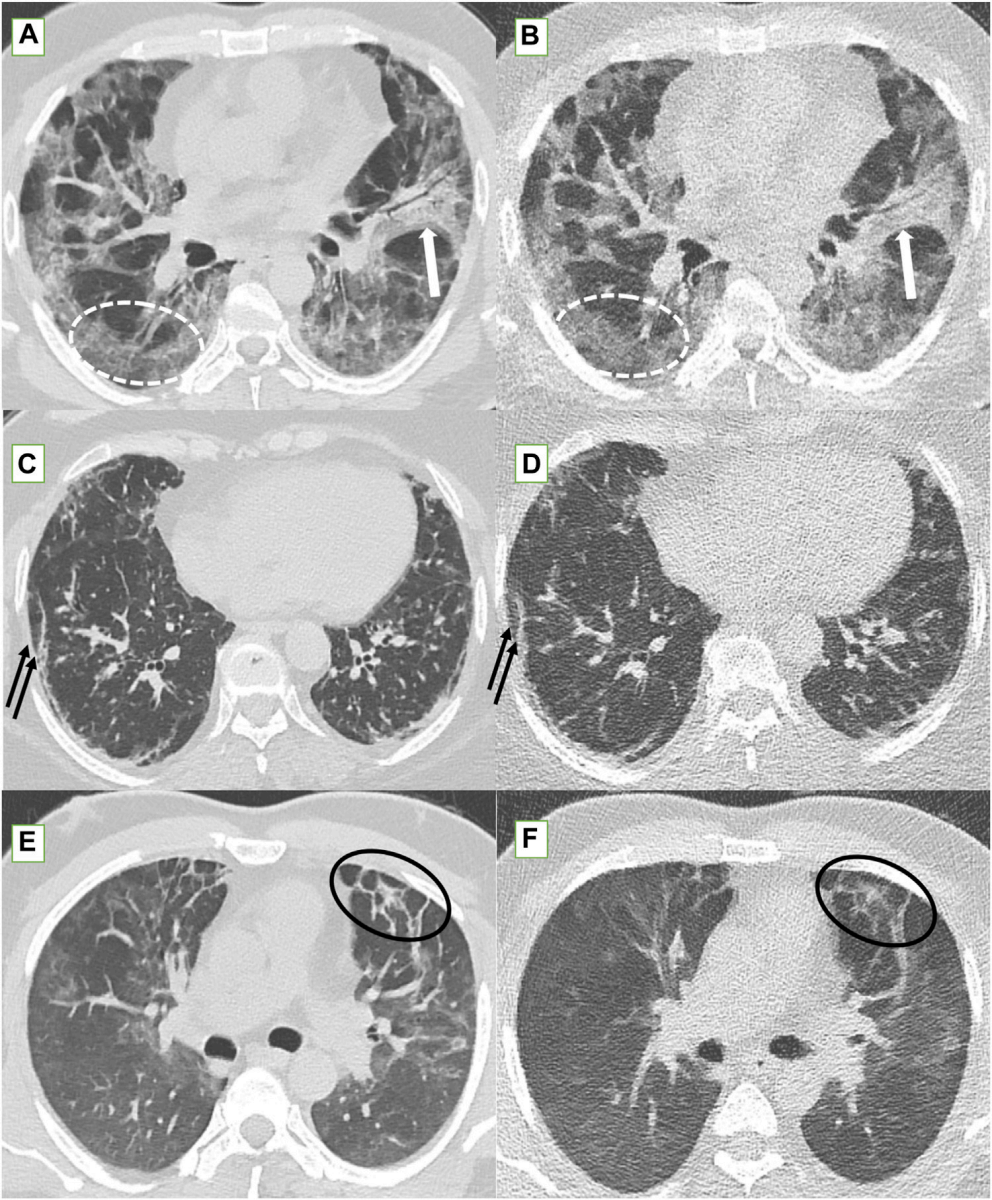
Standard-dose CT (SDCT) chest (left column) and corresponding ultra-low dose CT (ULDCT) chest images (right column) in three different patients: **(A,B)** 56-years old male long COVID patient with persistent cough and dyspnea. SDCT chest **(A)** and corresponding ULDCT chest **(B)** showing patchy GGOs (dotted white elliptical circle) in both lungs and patchy consolidation (white block arrow) in left upper lobe. Effective radiation dose for SDCT and ULDCT was 3.8 mSv and 0.24 mSv respectively. **(C,D)** 63-years old female long COVID follow up patient presented with dyspnea, and cough. SDCT chest **(C)** and corresponding ULDCT chest **(D)** showing subpleural linear opacities (black arrows) in both lungs. Effective radiation dose for SDCT and ULDCT was 4.8 mSv and 0.23 mSv respectively. **(E,F)** Another 43-years old male long COVID follow up patient presented with dyspnea. SDCT chest **(E)** and corresponding ULDCT chest **(F)** showing areas of inter-lobular septal thickening (black elliptical circle) in both lungs. Effective radiation dose for SDCT and ULDCT was 4.72 mSv and 0.26 mSv respectively. CT severity score calculated on both SDCT and ULDCT was similar in all three patients.

**FIGURE 4:**
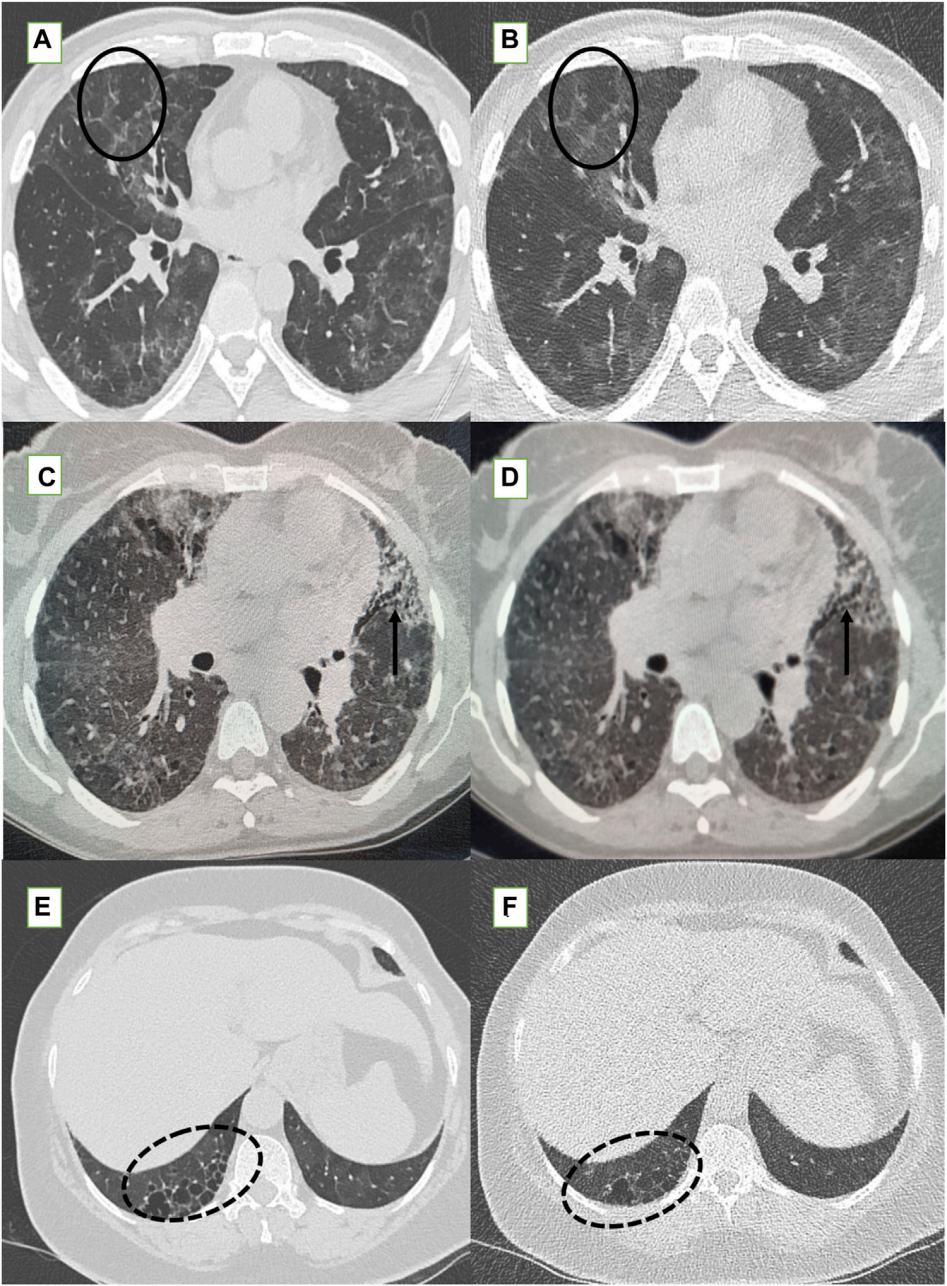
Standard-dose CT (SDCT) chest (left column) and corresponding ultra-low dose CT (ULDCT) chest images (right column) in three different patients: **(A,B)** 47-years old male long COVID follow up patient presented with fatigue and cough. SDCT chest **(A)** and corresponding ULDCT chest **(B)** showing patchy GGOs, septal thickening and mosaic attenuation (black elliptical circle) in both lungs. Effective radiation dose for SDCT and ULDCT was 4.3 mSv and 0.26 mSv respectively. **(C,D)** Another 56-years old female long COVID follow up patient presented with cough. SDCT chest **(C)** and corresponding ULDCT chest **(D)** showing tractional bronchiectasis (small black arrows) along with changes of sub-pleural cysts. Effective radiation dose for SDCT and ULDCT was 4.7mSv and 0.23 mSv respectively. **(E,F)** A 56-years old female long COVID follow up patient presented with dyspnea and cough. SDCT chest **(E)** and corresponding ULDCT chest **(F)** showing honeycombing in RLL (dotted black elliptical circle). Effective radiation dose for SDCT and ULDCT was 3.9 mSv and B-0.22 mSv respectively. CT severity score calculated on both SDCT and ULDCT was similar in all three patients.

**FIGURE 5:**
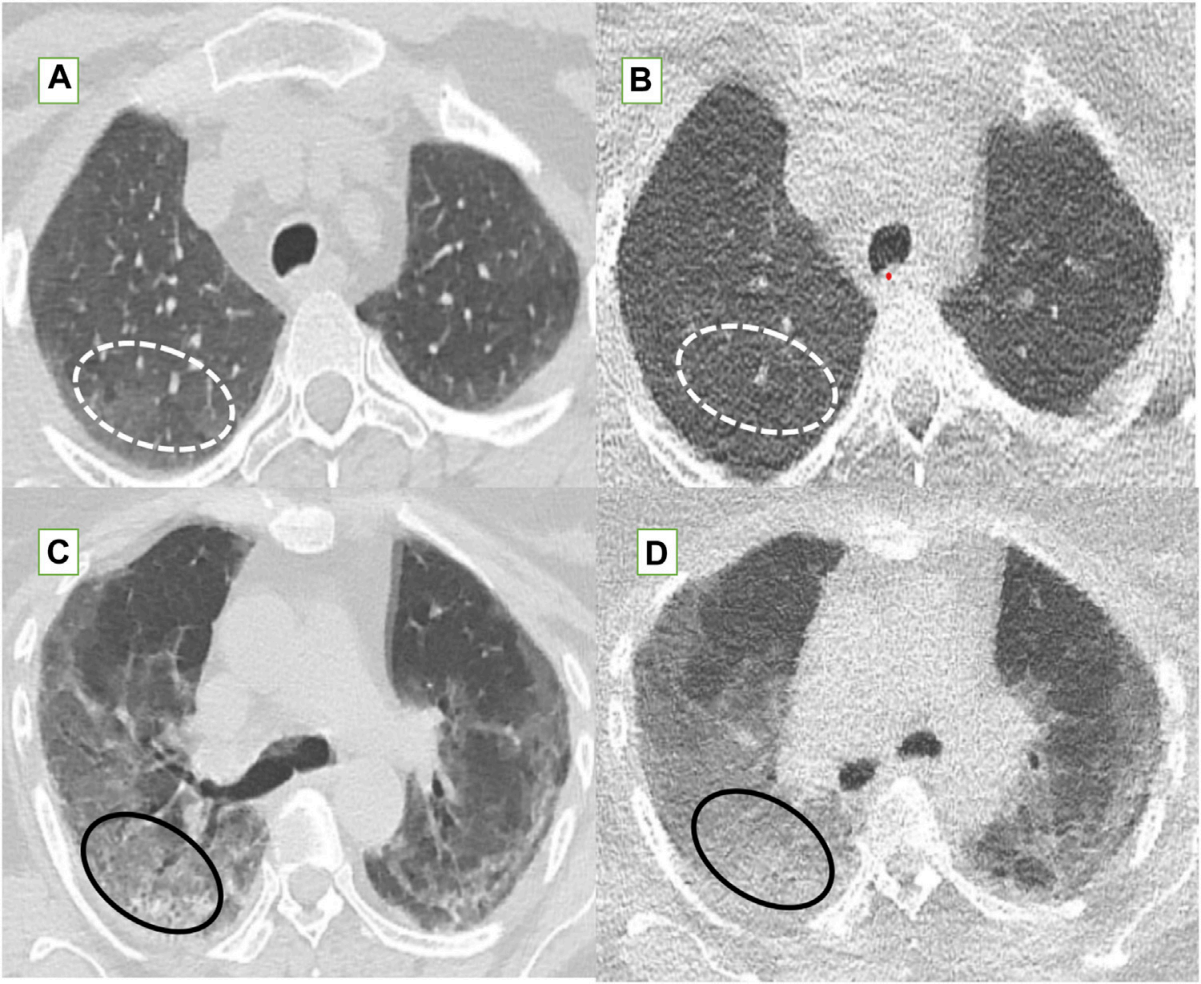
The fallacies of ultra-low dose CT (ULDCT) chest (right column) compared to standard dose CT (SDCT) chest (left column) have been depicted. **(A,B)** In a 46-year-old class 2 obese patient, SDCT **(A)** showed GGOs (dotted white elliptical circle) which were completely missed on the corresponding ULDCT images **(B)**. **(C,D)** In another 53-year-old class 3 obese patient, the SDCT chest **(C)** showed GGOs (black elliptical circle) which were interpreted as consolidation on the corresponding ULDCT images **(D)**.

The diagnostic performance of ULDCT (taking SDCT as a reference standard) for detecting the imaging patterns in long COVID has been elaborated in Table 2. The sensitivity of ULDCT for the detection of GGOs, consolidation, septal thickening/reticulation, atelectatic/parenchymal bands, bronchiectasis, and nodules were 72.7% (61.4%–82.3%), 100% (79.4%–100.00%), 71.6% (59.3%–82%), 81% (69.1%–89.8%), 72.2% (46.5%–90.3%), and 76.9% (56.4%–91.0%) respectively (*p* < 0.0001). The specificity of ULDCT for detection of consolidation was 89.3% (80.6%–95%). For the rest of the imaging patterns, specificity was 100% (*p* < 0.0001). The strength of agreement for GGOs was moderate (0.551), while it was substantial for consolidation (0.727), septal thickening/reticulations (0.625), and atelectatic/parenchymal bands (0.759). For the rest of the imaging patterns, the strength of agreement was excellent (0.81–1). Figures 3–5 show the representative lung abnormalities in long COVID as seen on SDCT and ULDCT.

**TABLE 2:** Diagnostic performance of ULDCT in detecting the imaging patterns of long COVID (with SDCT as the reference standard).

Imaging patterns	TP	TN	FP	FN	Sensitivity (95% CI)	Specificity (95% CI)	AUC (95% CI)	PPV (95% CI)	NPV (95% CI)	Diagnostic accuracy	Kappa	P-value
GGO	56	23	0	21	72.7% (61.4%–82.3%)	100% (85.2%–100%)	0.86 (0.78–0.92)	100% (93.6%–100%)	52.3% (36.7%–67.5%)	79% (70.9%–87.1%)	0.551	<0.0001
Consolidation	16	75	9	0	100% (79.4%–100%)	89.3% (80.6%–95%)	0.95 (0.88–0.98)	64% (42.5%–82%)	100% (95.2%–100%)	91% (85.3%–96.7%)	0.727	<0.0001
Interlobular septal thickening/reticulation	48	33	0	19	71.6% (59.3%–82%)	100% (89.4%–100%)	0.86 (0.77–0.92)	100% (92.6%–100%)	63.5% (49%–76.4%)	81% (73.2%–88.8%)	0.625	<0.0001
Atelectatic/Parenchymal bands	51	37	0	12	81% (69.1%–89.8%)	100% (90.5%–100%)	0.9 (0.83–0.95)	100% (93%–100%)	75.5% (61.1%–86.7%)	88% (81.5%–94.5%)	0.759	<0.0001
Architectural distortion	14	82	0	4	77.8% (52.4%–93.6%)	100% (95.6%–100%)	0.89 (0.81–0.94)	100.00% (76.8%–100%)	95.4% (88.5%–98.7%)	96% (92.1%–99.9%)	0.852	<0.0001
Mosaic attenuation	10	86	0	4	71.4% (41.9%–91.6%)	100% (95.8%–100%)	0.86 (0.77–0.92)	100.00% (69.2%–100%)	95.6% (89%–98.8%)	96% (92.1%–99.9%)	0.811	<0.0001
Bronchiectasis	13	82	0	5	72.2% (46.5%–90.3%)	100% (95.6%–100%)	0.86 (0.78–0.92)	100% (75.3%–100%)	94.3% (87.1%–98.1%)	95% (90.7%–99.4%)	0.81	<0.0001
Bronchial wall thickening	7	91	0	2	77.8% (40%–97.2%)	100% (96%–100%)	0.89 (0.81–0.94)	100% (59%–100%)	97.9% (92.5%–99.7%)	98% (95.2%–100%)	0.864	<0.0001
Tree in bud sign	3	96	0	1	75% (19.4%–99.4%)	100% (96.2%–100%)	0.88 (0.79–0.93)	100% (29.2%–100%)	99% (94.4%–100%)	99% (97%–100%)	0.852	<0.0001
Nodules	20	74	0	6	76.9% (56.4%–91%)	100% (95.1%–100%)	0.88 (0.81–0.94)	100% (83.2%–100%)	92.5% (84.4%–97.2%)	94% (89.3%–98.7%)	0.831	<0.0001
Emphysema	3	96	0	1	75% (19.4%–99.4%)	100% (96.2%–100%)	0.88 (0.79–0.93)	100% (29.2%–100%)	99% (94.4%–100%)	99% (97%–100%)	0.852	<0.0001
Cavitation	6	94	0	0	100% (54.1%–100%)	100% (96.2%–100%)	1 (0.96–1.00)	100% (54.1%–100%)	100% (96.2%–100%)	100% (81.4%–100%)	1	<0.0001
Honeycombing	4	95	0	1	80% (28.4%–99.5%)	100% (96.2%–100%)	0.9 (0.82–0.95)	100% (39.8%–100%)	99% (94.3%–100%)	99% (97%–100%)	0.884	<0.0001
Sub pleural/lung cysts	13	84	0	3	81.3% (54.4%–96%)	100% (95.7%–100%)	0.91 (0.83–0.96)	100% (75.3%–100%)	96.6% (90.3%–99.3%)	97% (93.6%–100%)	0.879	<0.0001
Pleural effusion	3	97	0	0	100% (29.2%–100%)	100% (96.3%–100%)	1 (0.96–1.00)	100% (29.2%–100%)	100% (96.3%–100%)	100% (81.4%–100%)	1	<0.0001
Pleural thickening	11	87	0	2	84.6% (54.6%–98.1%)	100% (95.9%–100%)	0.92 (0.85–0.97)	100% (71.5%–100%)	97.8% (92.1%–99.7%)	98% (95.2%–100%)	0.905	<0.0001
Lymphadenopathy	23	77	0	0	100% (85.2%–100%)	100% (95.3%–100%)	1 (0.96–1.00)	100% (85.2%–100%)	100% (95.3%–100%)	100% (81.4%–100%)	1	<0.0001
Hydropneumothorax	2	98	0	0	100% (15.8%–100%)	100% (96.3%–100%)	1 (0.96–1.00)	100% (15.8%–100%)	100% (96.3%–100%)	100% (81.4%–100%)	1	<0.0001

Abbreviations: TP, true positive; TN, true negative; FP, false positive; FN, false negative; AUC = area under curve; PPV, positive predictive value; NPV, negative predictive value; SDCT, standard dose computed tomography; ULDCT, ultra low dose computed tomography; GGOs, Ground glass opacities.

**FIGURE 6:**
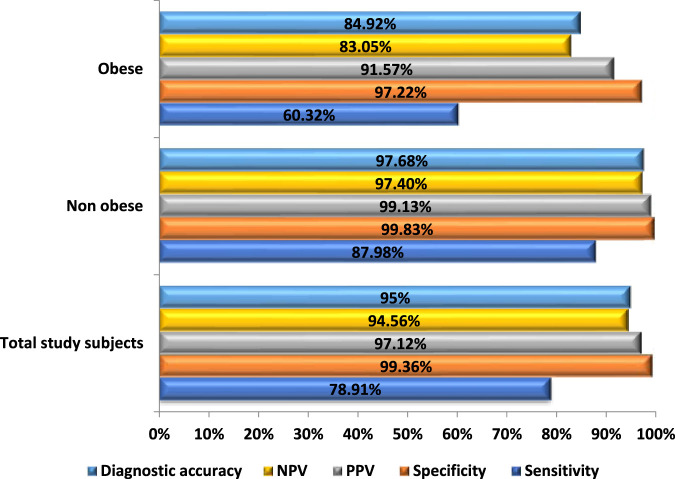
Bar diagram comparing the diagnostic performance of Ultra-low dose CT (ULDCT) chest in evaluation of imaging patterns of long COVID among subjects with BMI <30 kg/m^2^ and obese subjects, with standard-dose CT as the reference standard.

The diagnostic performance of ULDCT in “overall” and in the “obese” and “non-obese” groups has been depicted in Figure 6. The “overall” sensitivity, specificity, and diagnostic accuracy of ULDCT for detecting the imaging abnormalities of long COVID were 78.9% (74.5%–82.9%), 99.4% (98.8%–99.7%), and 95% (90.7%–99.4%) respectively. However, the sensitivity of ULDCT in “obese” patients was lower [60.3% (51.2%–68.9%)] compared to “non-obese” patients where it was 88% (83.4%–91.6%). The mean CT severity score for both SDCT and ULDCT was 9.7 ± 4.9 with a range of 1–18 for both the scans. Pearson’s correlation coefficient of r = 0.996 (*p* < 0.0001) (Supplementary Figure S1) was observed between CT severity scores for SDCT and ULDCT.

## Discussion

CT chest is an invaluable imaging tool to evaluate the sequelae of COVID-19 pneumonia and its complications [2, 5, 7, 29, 30]. However, with the enormous burden of COVID-19 recovered patients presenting with a prolonged course of respiratory illness, and many of them requiring frequent repeat scanning, the increased risk of radiation-induced health hazards always remains in these patients [10–13]. Thus, it became imperative to explore newer CT techniques like LDCT and ULDCT aimed at curtailing the radiation dose while still being able to achieve diagnostic quality images in keeping with the principle of “as low as reasonably achievable” (ALARA) [18–24]. Some authors have even attempted to study the utility of radiation-free imaging modalities like lung MRI, but it is still in the nascent stage with only anecdotal studies done in this direction [31, 32].

Our current study is a prospective study to evaluate the role of ULDCT chest in long COVID that has shown comparable image quality, sensitivity, specificity, and diagnostic accuracy of ULDCT to SDCT chest in non-obese subjects. The mean DLP for SDCT in our patients was 392.7 ± 140 mGy.cm, while for ULDCT, it was 18.2 ± 1.7 mGy.cm and we could achieve a net radiation dose reduction of 94.8% ± 1.7%. We acquired ULDCT at 80 kVp and 25 mA which is similar to the acquisition protocol used by Zarei F et al. [18] in another study evaluating the role of ULDCT in acute COVID-19 pneumonia.

The mean SNR of ULDCT recorded in our study was low (∼36% to that of SDCT), but the overall subjective image quality was comparable to that of SDCT in 80% of the ULDCT scans. These findings are in agreement with a study done by Samir et al. [21] who also reported a low SNR with ULDCT, but an acceptable quality for image interpretation.

The common imaging findings recorded in our study were GGOs (77%), interlobular septal thickening/reticulations (67%), atelectatic/parenchymal bands (63%), nodules (26%), and lymphadenopathy (23%). These findings align with the previous studies done on long COVID patients by various authors [2, 5, 7, 9, 33]. However, few patients in our cohort also showed hydropneumothorax, cavitation and tree-in-bud pattern, which can be attributed to the secondary bacterial or fungal infections [29, 30, 34]. The CT severity score calculated on SDCT and ULDCT in our cohort showed a very strong Pearson’s correlation coefficient of 0.996 (p-value < 0.0001), which is concurrent with the study done by Bahrami-Motlagh et al [20].

ULDCT in our study could not reliably depict GGOs in 21 subjects (completely missed in 12 patients and misinterpreted as consolidation in 9 patients). The sensitivity and diagnostic accuracy of ULDCT for detecting GGOs in our study were 72.7% and 79% respectively, which is in concordance with the study done by Zarei et al. [18] who reported a sensitivity of 62%, and diagnostic accuracy of 77%. However, ULDCT achieved a specificity of 100% in our study for detecting GGOs, which is significantly different from the study by Zarei et al. [18], who reported a specificity of 66%.

ULDCT also missed a few other abnormalities in some patients viz., septal thickening/reticulation (n = 19), atelectatic bands (n = 12), bronchiectasis (n = 5), and nodules (n = 6). However, ULDCT in our study achieved an overall diagnostic accuracy of 95% (90.7%–99.4%), which is in concordance with a previous comparative study done by Samir et al. [21] in acute COVID-19 pneumonia who reported similar diagnostic accuracy of ULDCT (90.38%–93.84%). Also, the overall specificity of ULDCT in detecting imaging correlates of long COVID was found to be high (99.4%) in our patient population. These findings suggest that ULDCT has the potential to become a valuable tool in monitoring the radiological progression in long COVID patients.

Another unique feature of our study was that we recorded the BMI of all participants and studied its effect on the image quality of ULDCT. We found that ULDCT scans in obese category patients (BMI ≥30 kg/m^2^) showed evidence of increased image graininess, increased artifacts, and reduced sharpness which compromised the overall diagnostic performance of ULDCT. Interestingly, this deterioration increased with increasing BMI and was more conspicuous in class II and class III categories of obese patients. In our study cohort, 7% of ULDCT scans showed high levels of image graininess, while blurring, and images of poor diagnostic confidence which were likely to misinterpret/miss imaging findings were seen in 9% each. Interestingly, only two ULDCT scans with compromised image quality belonged to non-obese patients, while all remaining scans in this category belonged to obese subjects. Sharp reproduction of segmental bronchi and lung parenchyma was not seen in 16 and 11 patients respectively, in the obese category. Artifacts were observed in 12 of our obese patients, while ULDCT scans showed high levels of image graininess and poor diagnostic confidence in 5 and 7 obese patients respectively. Likewise, of the 21 ULDCT scans in which GGOs were not detected, 13 of them belonged to the obese category. Furthermore, a statistically significant difference (p-value <0.0001) was observed in the diagnostic performance of ULDCT between obese and non-obese patients. In the obese group, sensitivity and diagnostic accuracy were demonstrably lower, reaching 60.3% (95% CI: 51.2%–68.9%) and 84.9% (95% CI: 77.8%–92.1%), respectively. Conversely, non-obese patients exhibited considerably higher sensitivity (88%, 95% CI: 83.4%–91.7%) and diagnostic accuracy (97.7%, 95% CI: 94.7%–100%). These results of ULDCT hold promise for the future clinical adoption of ULDCT, at least in the non-obese population group. Moreover, these results imply that the ULDCT scan protocol will require further modification (tweaking of the mAs or kVp) in obese patients to achieve desirable results. More research with larger patient cohorts is however required to further understand the effect of BMI on ULDCT scanning.

In the only study published on the utility of ULDCT in Long COVID by Wassipaul et al [25], the authors employed 100 kVp and 50 mAs as scan parameters, while we used 80 kVp and 25 mAs in our study. Despite utilizing comparatively higher scan parameters, Wassipaul’s study reported a lower median DLP (12.6 mGycm) and effective radiation dose (0.1764 mSv) compared to 18.2 mGycm and 0.25 mSv, respectively in our cohort. This can be due to the inherent dose reduction capabilities in some newer CT scanners, as we employed a Philips Brilliance iCT256, while Wassipaul’s utilized a dual-source Siemens Somatom Drive equipped with CAREdose. These findings emphasize the critical role of advanced CT systems with robust dose reduction technologies in minimizing radiation exposure without compromising image quality. However, despite a larger sample size in their study compared to ours (n = 153 vs. n = 100), all (100%) of our patients exhibited at least one relevant radiological finding, in contrast to only 29.4% in their study. This can be attributed to two factors - one, our study cohort included only the patients who were hospitalised with moderate to severe illness during their acute phase, and two the relatively older age group in our study population (52.9 ± 12.9 vs. 47.4 ± 15.3 years), both the recognised risk factors for long COVID [2, 35, 36]. However, our study demonstrated lower sensitivity (78.9% vs. 87.2%), potentially due to a higher obesity rate (21%) and use of lower kVp/mAs settings in our cohort, but it exhibited superior specificity (99.4% vs. 94.9%) and diagnostic accuracy (95% vs. 92.6%). Furthermore, the absence of a comprehensive qualitative (image noise, sharpness, artifacts, European image quality guidelines) and quantitative (including SNR) assessment, and non-inclusion of patient BMI, limits the impact of study by Wassipaul et al.

Despite the American Thoracic Society recommending against the use of ULDCT in ILD [37], we made an initial endeavour to evaluate its utility for assessing lung abnormalities in long COVID. Few other authors have also studied the utility of LDCT and ULDCT chest in the follow-up of patients with ILD with variable results. In a study by Hata et al. [38], it was found that the image quality of ULDCT with model-based iterative reconstruction (MBIR) was worse compared to SDCT, and detailed evaluation of ILD would not be possible with ULDCT. While, another study by Lim et al. [39] showed a comparable diagnostic performance of LDCT (with MBIR) to SDCT in the evaluation of ILDs, but they didn’t evaluate the utility of ULDCT. Since patients with long COVID can show findings similar to those seen in ILD like GGOs, septal thickening, parenchymal bands, etc. [2, 5, 7, 33]; more studies in long COVID patients using ULDCT are needed to substantiate our results.

There were a few limitations in our study. First, the single-center design with a relatively small sample size may have compromised the study’s statistical power. Second, even though we studied the imaging findings in long COVID patients comparing SDCT with ULDCT, we did not use any predefined follow-up timeline to scan the patients and this might have led to a varied spectrum of imaging findings. Third, the inclusion of patients with moderate to severe acute illness might have precluded the identification of more subtle lung abnormalities, potentially introducing bias. Likewise, we used same and fixed CT acquisition protocols for all the patients (both obese and non-obese categories) and that could have also affected the results in our study. Furthermore, there was lack of histopathological correlation to definitively confirm the nature of the observed imaging findings.

## Conclusion

In conclusion, the ULDCT chest has the potential to deliver acceptable diagnostic quality images in non-obese long COVID subjects at a much-reduced radiation exposure. The feasibility of ULDCT lies in the fact that it can be done on any CT machine by just changing the kVp and mAs without the need for any additional CT software or hardware. The accuracy of ULDCT can be further improved by taking into account the BMI of the patients and tweaking the scan acquisition protocols accordingly. However, to standardize and optimize the ULDCT protocol and validate its diagnostic accuracy, further multi-institutional research is needed amongst larger cohorts of patients.

## Summary Table

### What Is Known About This Subject?

• Long COVID patients experience persistent respiratory symptoms and require regular clinical and imaging follow-up.
• CT chest is imaging modality of choice for monitoring disease progression in long COVID patients, often requiring repeat scans.
• Exposure to ionizing radiation from repeated CTs can increase the risk of radiation-induced health effects.

### What Does This Paper Add?

• Comparison of diagnostic performance of ULDCT vs. SDCT chest in evaluation of imaging patterns of long COVID.
• ULDCT in non-obese subjects showed a significantly higher sensitivity and diagnostic accuracy compared to obese subjects, taking SDCT as the reference standard.
• ULDCT achieved a significant net radiation dose reduction (94.8% ± 1.7%) compared to SDCT.

## Concluding Statement

This work represents an advance in biomedical science because ULDCT demonstrated comparable diagnostic performance to SDCT in identifying lung abnormalities in long COVID, in non-obese individuals, with significantly reduced radiation dose.

## Data Availability

The raw data supporting the conclusions of this article will be made available by the authors, without undue reservation.

## Abbreviations

BMI, body mass index; COVID-19, Corona virus disease 2019; CT, Computed tomography; CTDIvol, Volume CT dose index; GGO, Ground glass opacities; ICRP, International commission on radiological protection; LDCT, Low dose CT; PACS, Post-Acute COVID-19 Syndrome; PASC, Post-Acute Sequelae of COVID-19; SDCT, Standard-dose CT; SNR, Signal to noise ratio; ULDCT, Ultra-low-dose CT.
