# Supplementary material for: Evaluating Lung Changes in Long COVID: Ultra-Low‐Dose vs. Standard‐Dose CT Chest
Source: Br J Biomed Sci. 2024 Sep 10;81:13385. doi: 10.3389/bjbs.2024.13385 (PMC11420527; doi:10.3389/bjbs.2024.13385)
Supplement: Supplementary file 1 [file DataSheet1.docx]

**Supplementary data**

**Supplementary tables**

**Table S1**: Detailed acquisition parameters of SDCT and ULDCT chest

| **Parameters** | **SDCT chest** | **ULDCT chest** |
| --- | --- | --- |
| Imaging system | 256-slice CT scanner (Philips Brilliance iCT256; Koninklijke Philips N.V., Netherlands) | |
| Scanning mode | Helical | Helical |
| Orientation | Head first | Head first |
| Extent of scan | Lung apices to domes of diaphragm | Lung apices to domes of diaphragm |
| Tube potential (kVp) | 120 | 80 |
| Tube current time (mAs) | AEC modulated | 25 |
| Tube current modulation technique | AEC | Fixed tube current |
| Pitch | 0.758 | 0.758 |
| Rotation time (s) | 0.5 | 0.5 |
| Slice thickness (in mm) | 0.625 | 0.625 |
| Iterative reconstruction technique | Hybrid iterative reconstruction software iDose level 6 | Hybrid iterative reconstruction software iDose level 6 |
| Reconstruction filter (lung window) | Lung enhanced (L) | Lung enhanced (L) |
| Reconstruction filter (mediastinal window) | Standard (B) | Standard (B) |

Abbreviations: CT = computed tomography, SDCT = Standard dose computed tomography, ULDCT = Ultra low dose computed tomography, AEC = Automated exposure control.

**Table S2**: Comparison of dose indices and image quality between SDCT and ULDCT

| **Dose indices and image quality** | | **SDCT** | **ULDCT** | | |
| --- | --- | --- | --- | --- | --- |
| CTDIvol (mGy) | | 10.78 ± 3.83 (mean ± S.D) (*p*<0.0001) | 0.5±0 (mean ± S.D) (*p*<0.0001) | | |
| DLP (mGycm) | | 392.68 ± 140.04 (mean ± S.D) (*p*<0.0001) | 18.19 ± 1.72 (mean ± S.D) (*p*<0.0001) | | |
| Effective radiation dose (mSv) | | 5.5 ± 1.96 (mean ± S.D) (*p*<0.0001) | 0.25 ± 0.02 (mean ± S.D) (*p*<0.0001) | | |
| Net effective radiation reduction | | 94.84 ± 1.69% (mean ± S.D) (*p*<0.0001) | | | |
| SNR | | 31.39 ± 5.49 (mean ± S.D) | 11.3 ± 4.62  (mean ± S.D) | N | 11.1 ± 4.46 |
|  |  |  |  | O | 10.32 ± 5.47 |
| Image graininess | No or minimum | 100 (100%) | 82 (82%) | N | 73 (73%) |
|  |  |  |  | O | 9 (9%) |
|  | Low | 0 (0%) | 11 (11%) | N | 4 (4%) |
|  |  |  |  | O | 7 (7%) |
|  | High, likely to misinterpret/miss imaging findings | 0 (0%) | 7 (7%) | N | 2 (2%) |
|  |  |  |  | O | 5 (5%) |
| Sharpness | Sharp | 100 (100%) | 81 (81%) | N | 73 (73%) |
|  |  |  |  | O | 8 (8%) |
|  | Average | 0 (0%) | 10 (10%) | N | 4 (4%) |
|  |  |  |  | O | 6 (6%) |
|  | Blur | 0 (0%) | 9 (9%) | N | 2 (2%) |
|  |  |  |  | O | 7 (7%) |
| Artifact | Absent | 96 (96%) | 87 (87%) | N | 78 (78%) |
|  |  |  |  | O | 9 (9%) |
|  | Artifacts present but not likely to misinterpret/miss imaging findings | 4 (4%) | 10 (10%) | N | 1 (1%) |
|  |  |  |  | O | 9 (9%) |
|  | Artifacts present and likely to misinterpret/miss imaging findings | 0 (0%) | 3 (3%) | N | 0 (0%) |
|  |  |  |  | O | 3 (3%) |
| Diagnostic confidence for labelling imaging finding as present or absent | Excellent | 100 (100%) | 80 (80%) | N | 73 (73%) |
|  |  |  |  | O | 7 (7%) |
|  | Moderate | 0 (0%) | 11 (11%) | N | 4 (4%) |
|  |  |  |  | O | 7 (7%) |
|  | Poor | 0 (0%) | 9 (9%) | N | 2 (2%) |
|  |  |  |  | O | 7 (7%) |

Abbreviations: SDCT = Standard dose computed tomography, ULDCT = Ultra low dose computed tomography, CTDI_vol_ = Volume CT dose index, DLP = dose-length product, S.D = standard deviation, SNR = signal to noise ratio, N=non-obese, O= obese

**Table S3:** Evaluation of image quality of CT chest as per European guidelines^26^

| **Visualisation of:** | **SDCT (n=100)** | **ULDCT (n=100)** | **P value** |
| --- | --- | --- | --- |
| Entire thoracic wall | 100 (100%) | 100 (100%) | 1^*^ |
| Entire thoracic aorta and vena cava | 100 (100%) | 100 (100%) | 1^*^ |
| Entire heart | 100 (100%) | 100 (100%) | 1^*^ |
| Entire lung parenchyma | 100 (100%) | 100 (100%) | 1^*^ |
| **Sharp reproduction of:** | **SDCT (n=100)** | **ULDCT (n=100)** |  |
| Thoracic aorta | 100 (100%) | 100 (100%) | 1^*^ |
| Anterior mediastinal structures, including thymic residue (if present) | 100 (100%) | 100 (100%) | 1^*^ |
| Trachea and main bronchi | 100 (100%) | 92 (92%) | 0.008^*^ |
| Paratracheal tissue | 100 (100%) | 100 (100%) | 1^*^ |
| Carina and lymph node area | 100 (100%) | 100 (100%) | 1^*^ |
| Oesophagus | 100 (100%) | 100 (100%) | 1^*^ |
| Pleuromediastinal border | 100 (100%) | 86 (86%) | 0.0001^*^ |
| Large and medium sized pulmonary vessels | 100 (100%) | 100 (100%) | 1^*^ |
| Segmental bronchi | 100 (100%) | 84(84%) | <0.0001^*^ |
| Lung parenchyma | 100 (100%) | 89 (89%) | 0.001^*^ |
| Border between the pleura and the thoracic wall | 100 (100%) | 85 (85%) | 0.0001^*^ |

^*^McNemar test

Abbreviations: SDCT = Standard dose computed tomography, ULDCT = Ultra-low dose computed tomography

**Supplementary figures**

**
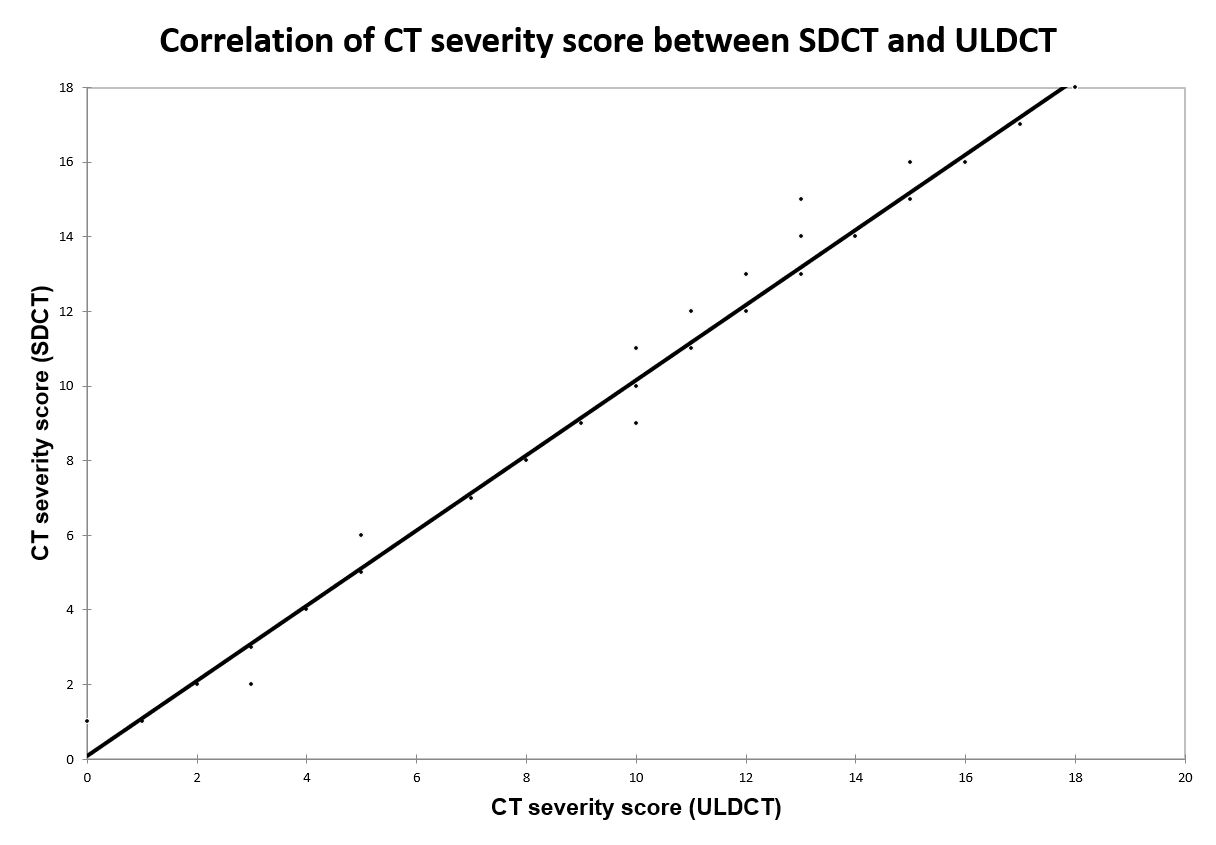
**

**Fig. S1: Scatter plot showing correlation of CT severity score between SDCT and ULDCT. Pearson correlation coefficient of r=0.996 (p<0.0001) was obtained.**
